# A six-gene prognostic model predicts overall survival in bladder cancer patients

**DOI:** 10.1186/s12935-019-0950-7

**Published:** 2019-09-05

**Authors:** Liwei Wang, Jiazhong Shi, Yaqin Huang, Sha Liu, Jingqi Zhang, Hua Ding, Jin Yang, Zhiwen Chen

**Affiliations:** 1 Urology Institute of People’s Liberation Army, Southwest Hospital, Third Military Medical University (Army Medical University), Chongqing, 400038 People’s Republic of China; 2 Unit 32357 of People’s Liberation Army, Pujiang, 611630 People’s Republic of China; 3 Department of Cell Biology, Third Military Medical University (Army Medical University), Chongqing, 400038 People’s Republic of China

**Keywords:** Bladder cancer, Methylation, TCGA, LASSO Cox, Nomogram, Survival analysis

## Abstract

**Background:**

The fatality and recurrence rates of bladder cancer (BC) have progressively increased. DNA methylation is an influential regulator associated with gene transcription in the pathogenesis of BC. We describe a comprehensive epigenetic study performed to analyse DNA methylation-driven genes in BC.

**Methods:**

Data related to DNA methylation, the gene transcriptome and survival in BC were downloaded from The Cancer Genome Atlas (TCGA). MethylMix was used to detect BC-specific hyper-/hypo-methylated genes. Metascape was used to carry out gene ontology (GO) enrichment and Kyoto Encyclopedia of Genes and Genomes (KEGG) pathway analyses. A least absolute shrinkage and selection operator (LASSO)-penalized Cox regression was conducted to identify the characteristic dimension decrease and distinguish prognosis-related methylation-driven genes. Subsequently, we developed a six-gene risk evaluation model and a novel prognosis-related nomogram to predict overall survival (OS). A survival analysis was carried out to explore the individual prognostic significance of the six genes.

**Results:**

In total, 167 methylation-driven genes were identified. Based on the LASSO Cox regression, six genes, i.e., ARHGDIB, LINC00526, IDH2, ARL14, GSTM2, and LURAP1, were selected for the development of a risk evaluation model. The Kaplan–Meier curve indicated that patients in the low-risk group had considerably better OS (P = 1.679e−05). The area under the curve (AUC) of this model was 0.698 at 3 years of OS. The verification performed in subgroups demonstrated the validity of the model. Then, we designed an OS-associated nomogram that included the risk score and clinical factors. The concordance index of the nomogram was 0.694. The methylation levels of IDH2 and ARL14 were appreciably related to the survival results. In addition, the methylation and gene expression-matched survival analysis revealed that ARHGDIB and ARL14 could be used as independent prognostic indicators. Among the six genes, 6 methylation sites in ARHGDIB, 3 in GSTM2, 1 in ARL14, 2 in LINC00526 and 2 in LURAP1 were meaningfully associated with BC prognosis. In addition, several abnormal methylated sites were identified as linked to gene expression.

**Conclusion:**

We discovered differential methylation in BC patients with better and worse survival and provided a risk evaluation model by merging six gene markers with clinical characteristics.

## Background

Bladder cancer (BC) is one of the most difficult to treat and costly cancers due to its relapse tendency and chemoresistance [1]. In total, 76,000 new cases and 16,000 deaths are attributed to BC in the USA per year [2]. With such a large patient population, accurately diagnosing and effectively treating BC have become difficult challenges for basic medical researchers and urologists.

Epigenetic dysregulation is an important mechanism of tumorigenesis that affects the expression of numerous genes [3]. Aberrant DNA methylation, i.e., hyper- or hypomethylation, on CpG islands of promoters is one such mechanism, resulting in aberrant gene expression and having a major impact on the biological behaviour of BC [4, 5]. DNA methylation could also serve as a good biomarker for clinical diagnosis because of its stable and easily detectable attributes in many types of clinical specimen [6, 7]. Dulaimi et al. [8] reported that the detection of hypermethylation in the APC, RASSF1A, and ARF genes in BC patients may act as a non-invasive method for early diagnosis. Casadio et al. [9] also indicated that the methylation frequencies of HIC1, GSTP1 and RASSF1A could predict BC recurrence. Ohad et al. [10] found that CDH13 is downregulated by promoter methylation in BC patients, and this may be closely associated with tumour development.

The TCGA project aims to catalogue and discover major molecular changes to create a comprehensive “map” of the human cancer genome [11]. The multiple dimensions of data and massive samples not only provide a more comprehensive view of cancer but also enable the finding of better biomarkers, which could affect cancer treatment and prognosis [12]. DNA methylation data are also included in the massive data set, and a computational protocol called MethylMix can distinguish disease-specific hyper/hypomethylation genes, both of which are publicly available [13]. Several studies have been conducted to assess methylation-driven genes using the MethylMix algorithm and TCGA database [13–15].

In this study, we identified BC-related methylation-driven genes by using the data from the TCGA database. By coupling DNA methylation and gene transcriptome data, we identified methylation-driven genes and further constructed a model of DNA methylation status to predict prognosis in BC patients.

## Materials and methods

### Data processing and analysis

We downloaded DNA methylation, gene transcriptome and clinical survival data of BC patients from TCGA [16]. There were 437 samples with DNA methylation data (21 normal and 416 cancer), 430 samples with gene transcriptome data (19 normal and 411 cancer), and 404 patients with valid survival data. These data are an open resource, and no ethical issues were involved.

First, we applied the Limma package in R to extract the DNA methylation data. Next, we used the edgeR package to obtain the gene expression data. A comprehensive analysis was performed to obtain the following three data matrices: DNA methylation (normal, cancer) and gene expression. Subsequently, we used MethylMix [13, 17] to compare DNA methylation of cancer with that of normal tissue to detect specific genes, particularly BC-specific hyper/hypomethylation genes, and the methylation level of these genes was described as ‘transcriptionally predictive’. A mixture model of each gene was built, and Wilcoxon rank tests were computed with the following parameters: set as logFC > 0, P < 0.01, and Cor < − 0.3.

### Functional enrichment and pathway analysis

Metascape [18] integrates several authoritative data resources, such as GO, KEGG, UniProt and DrugBank, so that it can execute pathway enrichment and biological process annotation to provide comprehensive and detailed information for each gene [19]. GO enrichment and KEGG pathway analyses of the genes identified by MethylMix were performed. Only terms with P < 0.01, a minimum count of 3 and an enrichment factor > 1.5 were considered significant.

### Construction of the risk assessment model

First, the genes identified by MethylMix were applied to a univariate Cox regression. Second, we used a LASSO regression to narrow the range of target genes because the predictor variable was much larger than the sample content in the gene expression data. A strong correlation often exists between the variables, which is suggestive of high dimensionality and collinearity, and this method could decrease the characteristic dimension [20]. Then, we built a multivariate Cox regression model to select the genes that were most tightly associated with survival [21]. In addition, we validated this model in subgroups based on different characteristics. The following 12 subgroups based on different clinical characteristics and 9 subgroups based on different mRNA subtypes and mutational signatures [5] were subjected to further tests: high grade (n = 381), low grade (n = 20), stage I (n = 2), stage II (n = 128), stage III (n = 139), stage IV (n = 133), muscle-invasive (n = 368), non-muscle-invasive (n = 4), no distant metastasis (n = 193), distant metastasis (n = 11), lymph node metastasis (n = 169), no lymph node metastasis (n = 235), or Msig 1 (n = 28), Msig 2 (n = 220), Msig 3 (n = 99), Msig 4 (n = 55), basal squamous (n = 137), luminal (n = 26), luminal infiltrated (n = 77), luminal papillary (n = 140), and neuronal (n = 20).

The sensitivity and specificity of the model in the diagnosis of BC were analysed by a time-dependent ROC curve.

Furthermore, an OS-associated nomogram including the risk score and clinical factors was designed using the rms [22] and the Hmisc [23] packages in R. Calibration curves were drawn, and the concordance index (C-index) was computed to assess the efficiency of the nomogram.

### Survival analysis

Kaplan–Meier curves were used to distinguish the connection between these genes and prognosis. A subgroup analysis was performed by dividing the patients based on clinical characteristics. A methylation/methylation site and gene expression matched survival analysis was carried out to explore the prognostic significance of these genes individually. The relationships between gene expression and methylated sites were additionally examined.

### Data processing

All data analyses were performed with R [24]. Student’s t-test was used to evaluate the differences between two groups. The log-rank test was applied in the Kaplan–Meier survival examination.

## Results

### TCGA data acquisition and filtering methylation-driven genes

**Fig. 1:**
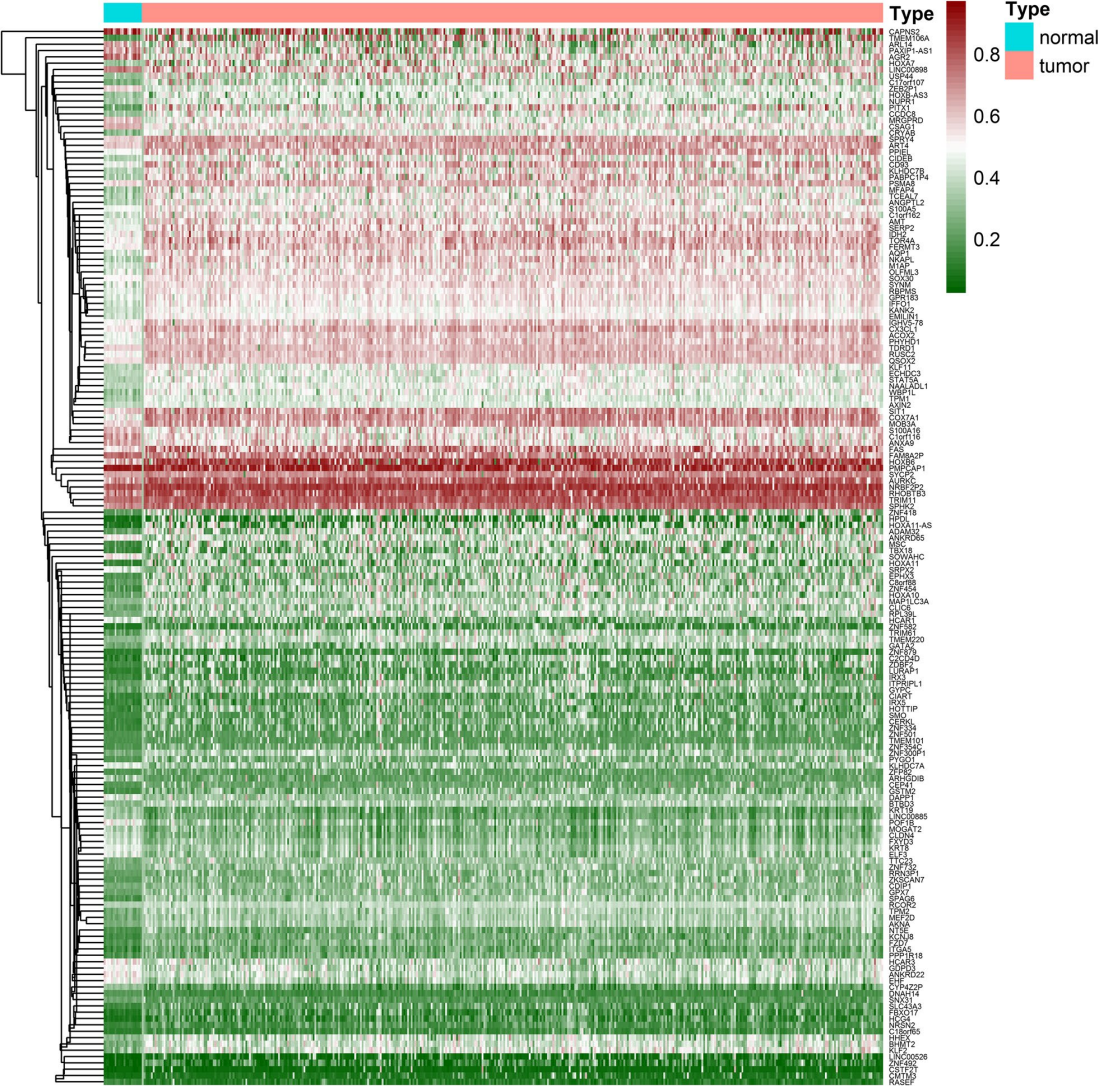
Heatmap of 167 BC-related methylation-driven genes. Red to green shows a trend from hypermethylation to hypomethylation

**Fig. 2:**
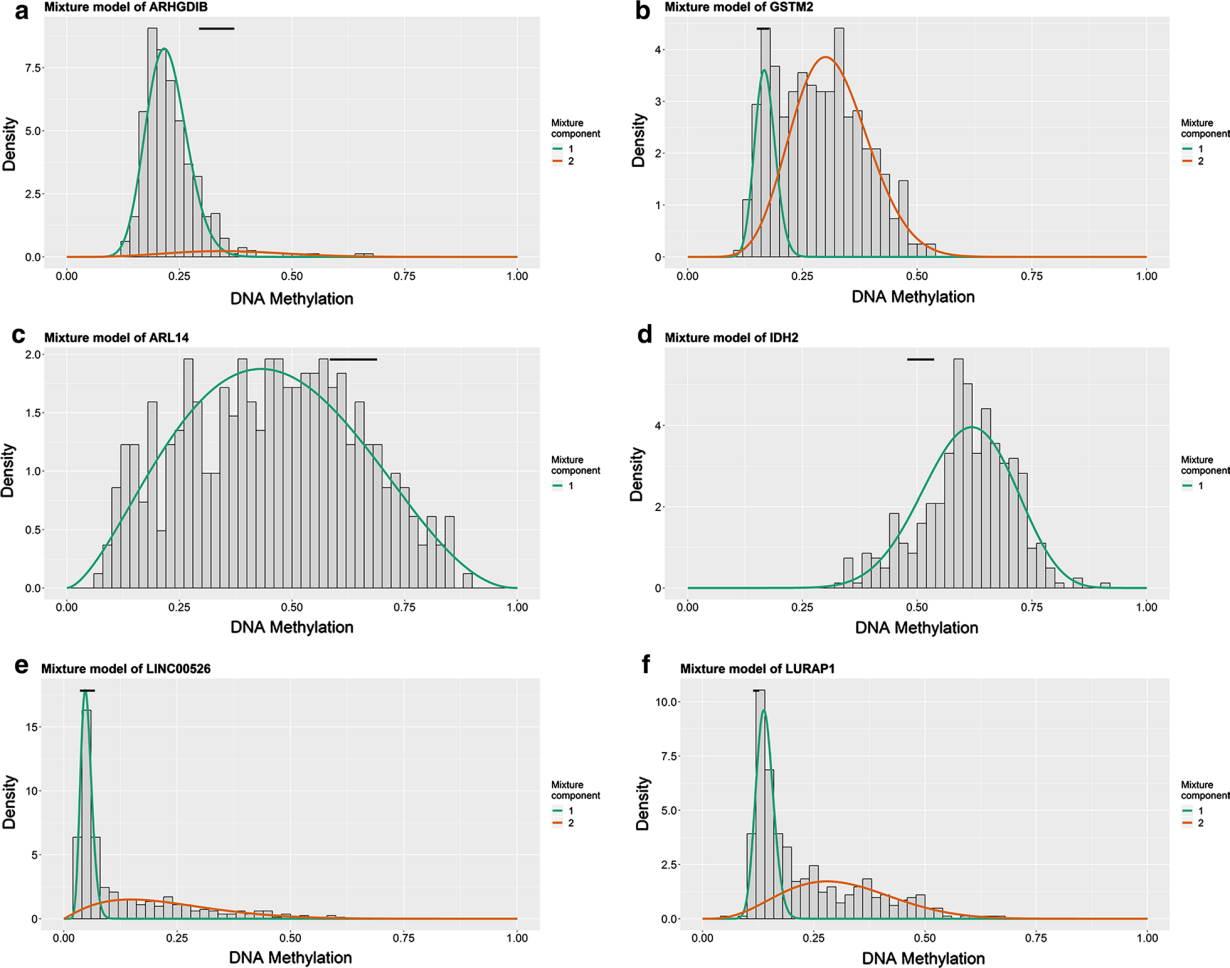
Mixture models of 6 of the 167 genes. The x-axis indicates the degree of methylation, the y-axis indicates the proportion at different degrees, the curve indicates the peak value, and the black bar indicates the normal methylation degree (**a**–**f**)

In total, 167 genes were identified (Fig. 1; Additional files 1, 2) by applying MethylMix to the three matrices, and a mixture model of each gene was constructed (Fig. 2). Intuitively, the relationship between the peak curve and the black bar indicates whether a gene is hyper- or hypomethylated.

### Functional enrichment and pathway analysis

**Fig. 3:**
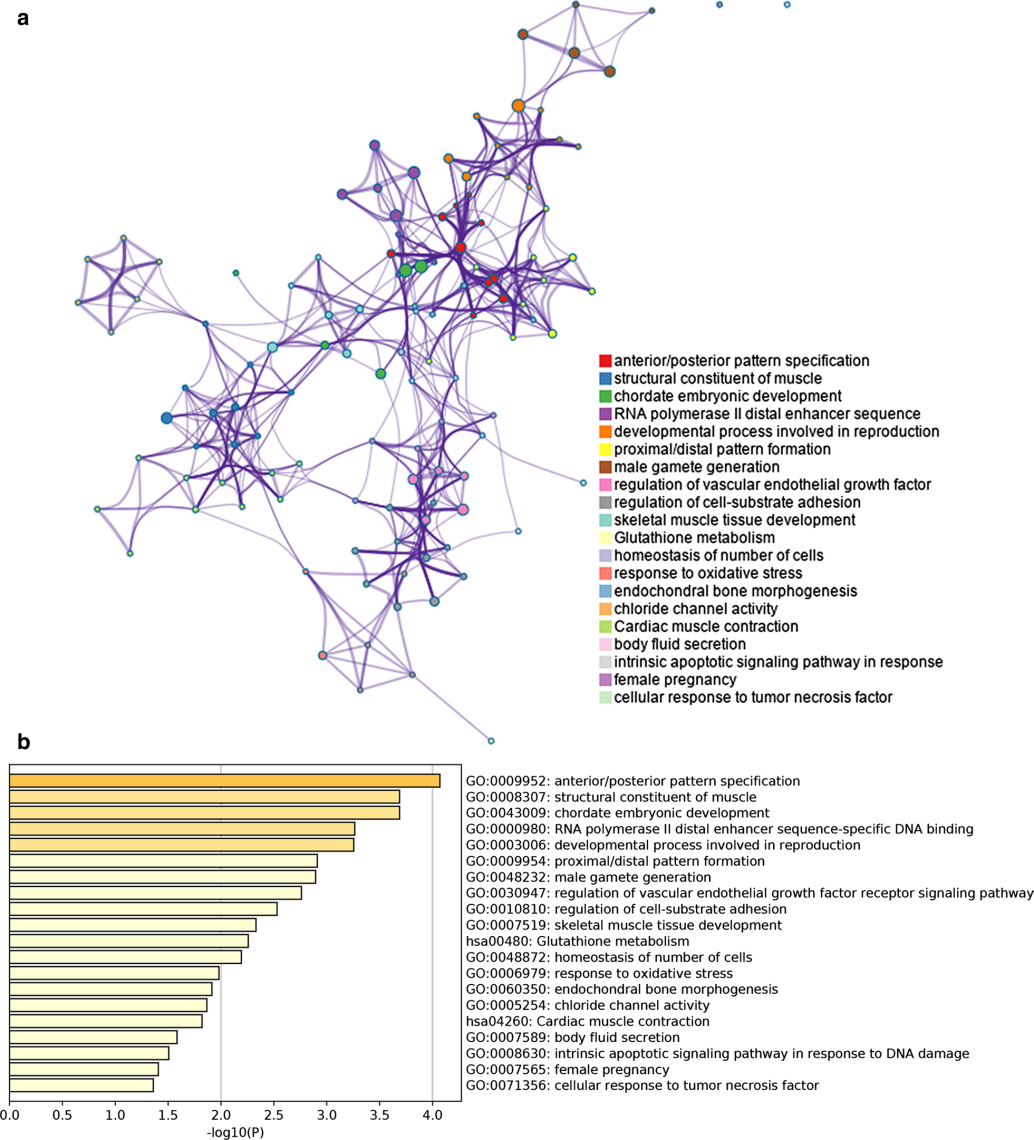
Metascape analysis. **a** Network of enriched sets coloured by ID. Threshold: 0.3 kappa score; similarity score > 0.3. **b** Heatmap coloured by P-values

The Metascape analysis shows the top 20 clusters of enriched sets (Fig. 3). These genes were enriched in the molecular function (MF) categories structural constituents of muscle and RNA polymerase II distal enhancer sequence-specific DNA binding. For biological process (BP), these genes showed enrichment in anterior/posterior pattern specification, chordate embryonic development, intrinsic apoptotic signalling pathway in response to DNA damage and so on (Additional file 3). The KEGG pathway data were enriched in Glutathione metabolism and Cardiac muscle contraction.

### Construction of the risk assessment model

**Fig. 4:**
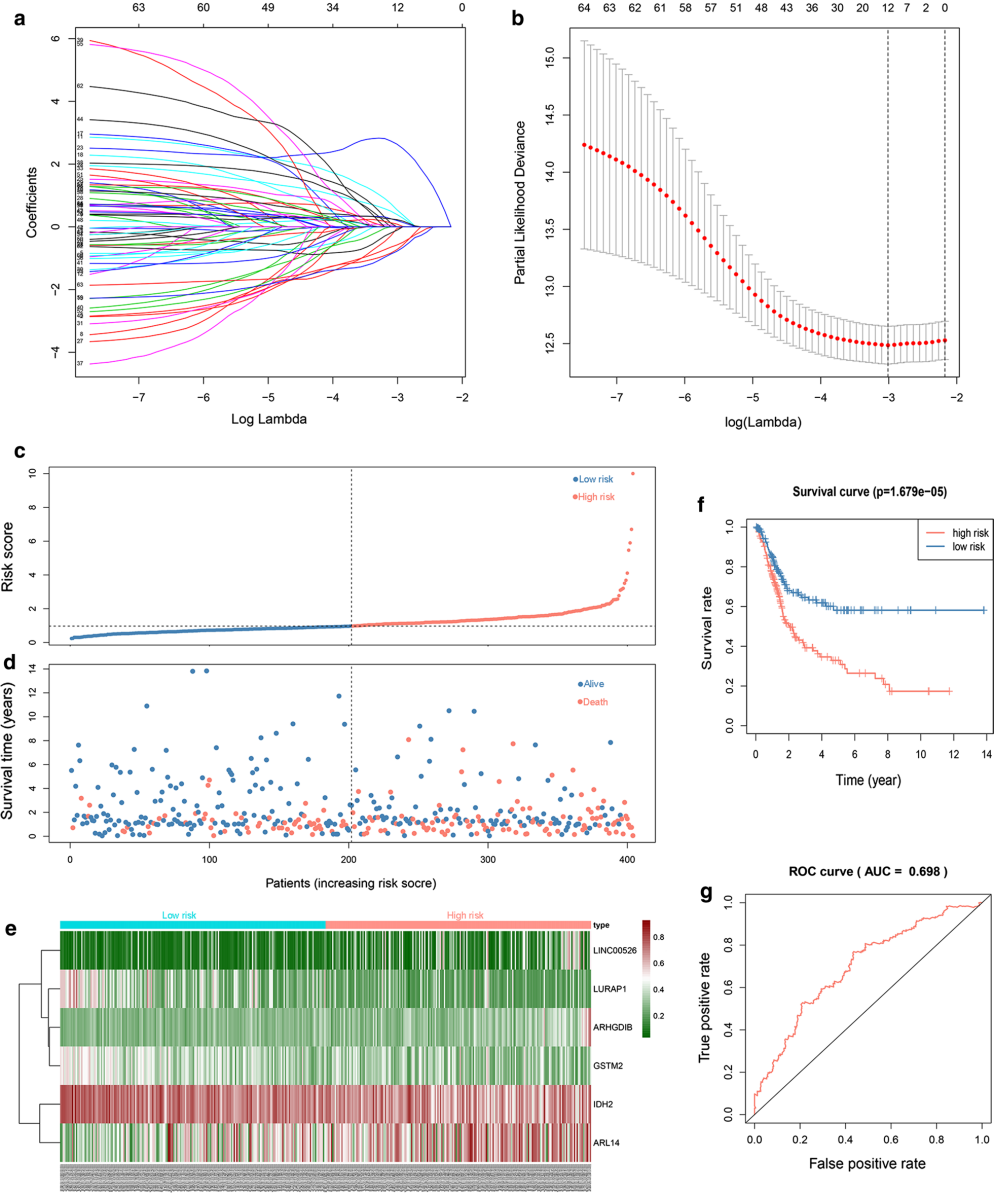
Identification of prognostic genes in BC patients. **a** LASSO coefficients. **b** Plots of the cross-validation error rates. The dashes signify the value of the minimal error and greater λ value. **c** Risk score distribution in the two groups. **d** Survival overview in the two groups. **e** Heatmap of six genes in the two groups. **f** Survival curve of the two groups. **g** Time-dependent ROC curve for 3-year survival prediction

The results of the univariate Cox regression analysis of 167 genes were used in the LASSO regression to identify robust markers. A set of twelve genes (DAPP1, TCEAL7, PAXIP1-AS1, TDRD1, NUPR1, ARHGDIB, LINC00526, IDH2, ARL14, KLHDC7A, GSTM2, and LURAP1) and their coefficients were computed (Fig. 4a, b). Then, multivariate Cox regression analyses were performed, and a six-gene model was constructed according to their methylation levels and coefficients. Risk score = (ARHGDIB * 4.533910954) + (LINC00526 * 1.999499891) + (IDH2 * − 2.048441591) + (ARL14 * 0.779318158) + (GSTM2 * − 1.375204374) + (LURAP1 * − 1.504186188).

The risk score of each BC patient was computed, and the patients were assigned to the low-risk (n = 202) or high-risk (n = 202) group based on the median cut-off value (Additional file 4, Fig. 4c). Intuitively, the number of deaths was significantly higher in the high-risk group (Fig. 4d). The distribution of the six genes across all samples showed that the patients in the low-risk group were likely to have a higher degree of methylation of IDH2, GSTM2 and LURAP1. In contrast, the patients in the high-risk group were inclined to have higher methylation of ARHGDIB, LINC00526, and ARL14 (Fig. 4e). The Kaplan–Meier analysis of all patients (Fig. 4f) indicated that the survival of the patients in the low-risk group was significantly better than that of the patients in the high-risk group (P = 1.679e−05). The AUC of the survival assessment model of the six methylation-driven genes was 0.698 at 3 years of OS (Fig. 4g).

**Fig. 5:**
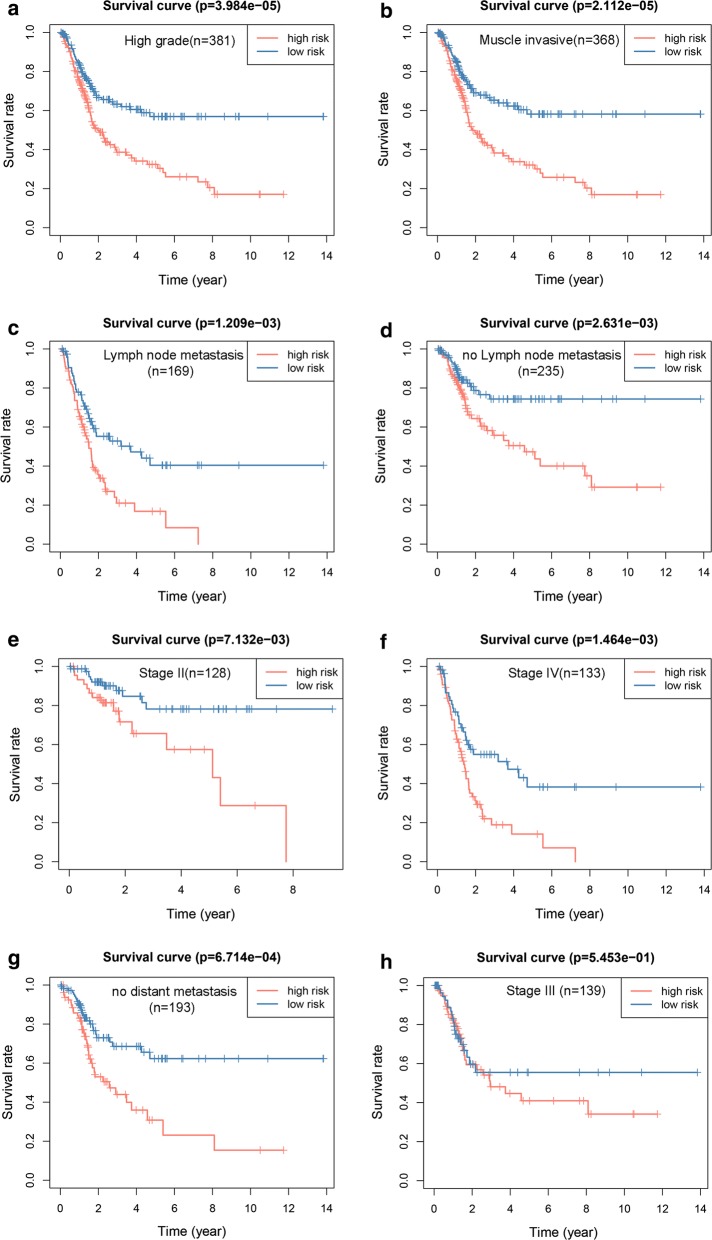
Kaplan–Meier survival curves. Validation of the six-gene model based on different clinical characteristics (**a**–**h**)

We further tested the survival assessment model by Kaplan–Meier analysis in subgroups. Of the 12 subgroups classified by clinical characteristics, there were no enough cases for stage I (n = 2), non-muscle-invasive (n = 4), and distant metastasis (n = 11), and all patients in the low grade group (n = 20) were alive, the test in the remaining 8 groups showed the same results as in all patients (Fig. 5a–h). Although the P-value in the stage III group was not statistically significant (Fig. 5h), these patients all showed the same predictive trends. Of the 9 subgroups (Additional file 4) classified by different mRNA subtypes or mutational signatures of BC [5]. The Kaplan–Meier curves (Additional file 5) show that this model is still effective in the Msig2, Msig3, luminal infiltrated, luminal papillary, and neuronal groups. Thus, the model has a certain reliability and practicability in evaluating prognosis.

### Establishment and evaluation of the nomogram

**Fig. 6:**
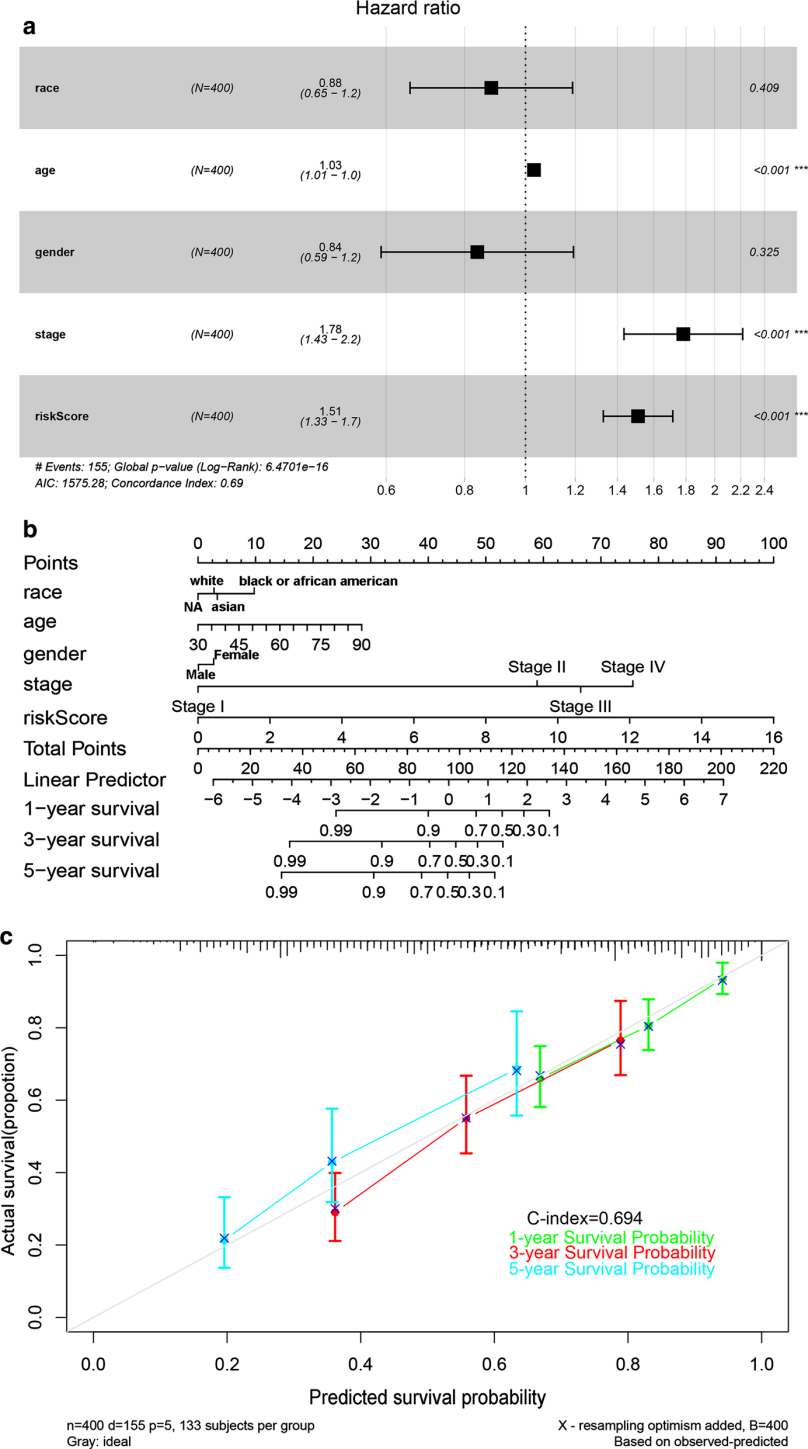
Six-gene model for survival prediction. **a** Multivariate Cox proportional hazard model of the risk score and clinical factors. **b** OS-associated nomogram. **c** Nomogram calibration plots. ***P < 0.001

We designed a nomogram to predict the survival probability of each patient. In the nomogram, each predictor was assigned a score. Based on the Cox analysis results (Table 1), six genes were integrated in the nomogram to predict the survival probability of BC patients (Additional file 6). The hypermethylation of ARHGDIB, LINC00526, and ARL14 is a risk factor for OS. Similarly, we carried out an analysis of the risk score and five clinical factors (Table 2; Fig. 6a). Based on the univariate Cox analysis, four factors (race, age, gender, and stage) and the risk scores were included in the multivariate Cox analysis (the factor ‘grade’ was not suitable for further analysis according to R). We constructed a nomogram to predict the OS probability (Fig. 6b). The C-index of this model was 0.694 (Fig. 6c). The predicted survival rate is close to the actual survival situation, and the prediction accuracy is similar to the ROC curve.

**Table 1:** Coefficients based on a Cox regression analysis of six genes

Variables	Univariate analysis			Multivariate analysis		
	HR	95% CI of HR	P-value	HR	95% CI of HR	P-value
ARHGDIB	76.13919	9.911825–584.8748	3.11E−05	93.12205	11.51496–753.0825	2.13E−05
LINC00526	3.310809	1.013776–10.8125	0.04741	7.385362	2.17362–25.09342	0.001355
IDH2	0.161273	0.031827–0.817183	0.027538	0.128936	0.024135–0.688822	0.016576
ARL14	3.002286	1.308697–6.887556	0.009459	2.179985	0.884152–5.375019	0.09054
GSTM2	0.088565	0.01622–0.483568	0.005128	0.252788	0.038877–1.643669	0.149947
LURAP1	0.145702	0.034246–0.619906	0.009128	0.222198	0.046774–1.055542	0.058494

*CI* confidence interval, *HR* hazard ratio

**Table 2:** Coefficients based on a Cox regression analysis of the risk score and clinical factors

Variables	Univariate analysis				Multivariate analysis			
	HR	95% CI of HR		P-value	HR	95% CI of HR		P-value
Race	1.13700	0.8596391–1.503856		0.368166	0.882196	0.654978–1.188243		0.409438
Age	1.03580	1.0185976–1.053294		3.85E−05	1.031468	1.014000–1.049236		0.000377
Gender	0.89812	0.6331828–1.273916		0.546846	0.837966	0.589105–1.191956		0.325475
Grade	961019	0 (Inf)		0.991439	–	–		–
Stage	1.86112	1.5099545–2.29397		5.80E−09	1.782751	1.434223–2.215973		1.90E−07
Risk score	1.58277	1.4006472–1.788583		1.81E−13	1.510365	1.329843–1.715391		2.16E−10

*CI* confidence interval, *HR* hazard ratio

### Prognostic assessment of methylation-driven genes

**Fig. 7:**
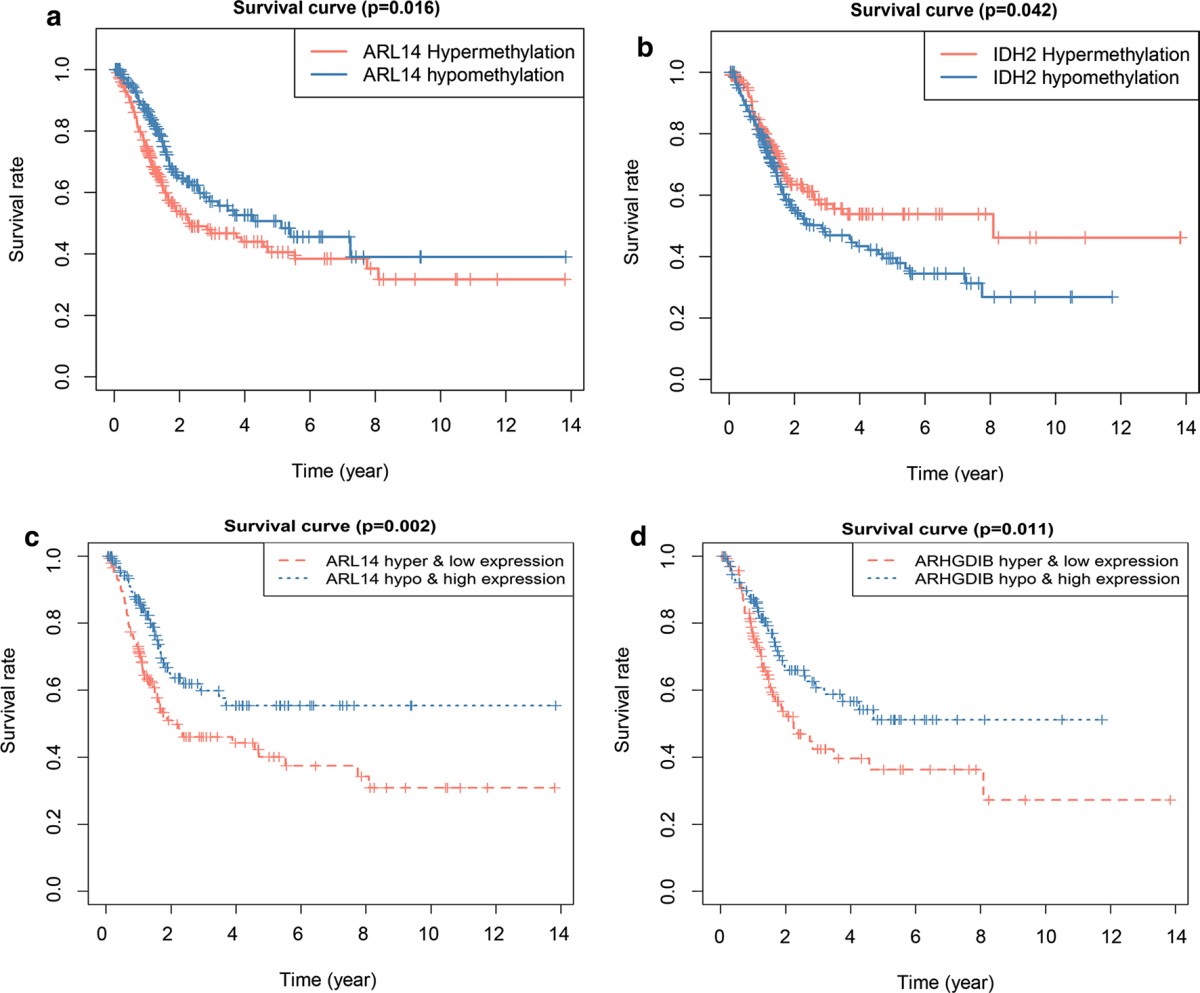
Kaplan–Meier survival curves. Hyper/hypomethylation analysis of ARL14 and IDH2 (**a**, **b**). Methylation and gene expression matched analysis of ARL14 and ARHGDIB (**c**, **d**)

**Fig. 8:**
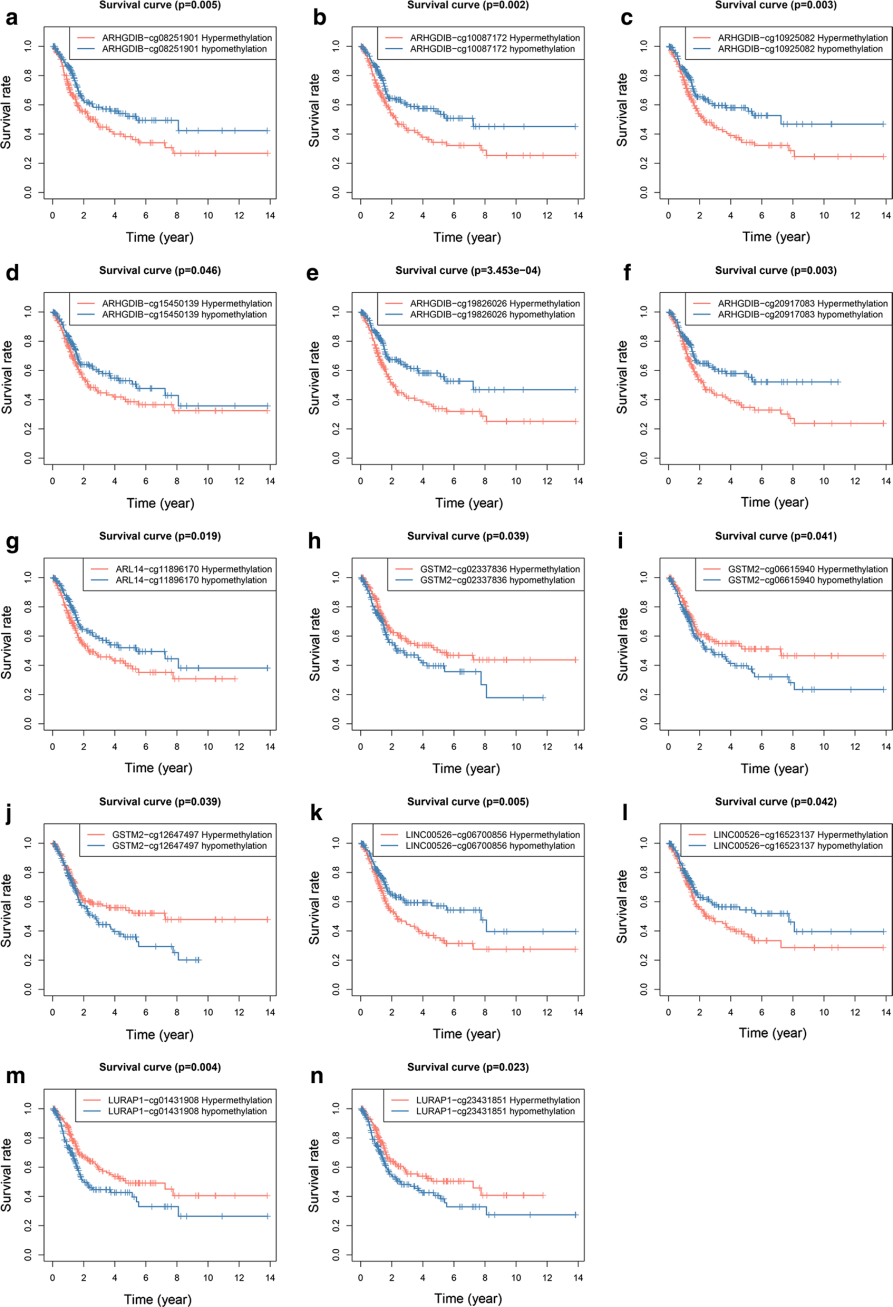
Kaplan–Meier survival curves. Six methylation sites in ARHGDIB (**a**–**f**). One methylation site in ARL14 (**g**). Three methylation sites in GSTM2 (**h**–**j**). Two methylation sites in LINC00526 (**k**, **l**). Two methylation sites in LURAP1 (**m**, **n**)

The survival status evaluation of 6 genes was computed by the Survival package in R. IDH2 and ARL14 were identified as independent prognostic indicators (Fig. 7a, b), and the hypomethylation of IDH2 and hypermethylation of ARL14 were related to worse prognosis in BC patients. The methylation/methylation-site and gene expression matched evaluation was additionally carried out to discover the prognostic value. We found that a high expression and hypomethylation of ARHGDIB and ARL14 were meaningfully correlated with better prognosis (Fig. 7c, d). Among the six genes, 6 methylated sites in ARHGDIB (Fig. 8a–f), 1 methylated site in ARL14 (Fig. 8g), 3 methylated sites in GSTM2 (Fig. 8h–j), 2 methylated sites in LINC00526 (Fig. 8k, l) and 2 methylated sites in LURAP1 (Fig. 8m, n) were significantly associated with BC prognosis. The hypermethylation of 5 sites in LURAP1 and GSTM2 is associated with better prognosis; in contrast, the hypermethylation of another 9 sites in ARHGDIB, LINC00526 and ARL14 is associated with poor prognosis. This result is consistent with the results shown in Figs. 4e and 5a. The hypermethylation of IDH2, LURAP1, and GSTM2 may act as a protective factor in BC patients. Other three genes, i.e., ARHGDIB, LINC00526, and ARL14, may have the opposite effect. Additionally, several abnormally methylated sites were identified as linked to gene expression (Table 3; Additional file 7).

**Table 3:** Correlation between gene expression and methylated sites

Gene and methylation site	Correlation	P-value
ARL14-cg20725880	− 0.686	5.651e−58
ARL14-cg24147596	− 0.547	2.919e−33
GSTM2-cg03942855	− 0.552	5.679e−34
GSTM2-cg12647497	− 0.586	4.89e−39
LINC00526-cg05390530	− 0.58	4.125e−38
LINC00526-cg10885961	− 0.619	1.348e−44
LINC00526-cg14291066	− 0.597	9.105e−41
LINC00526-cg15258847	− 0.571	1.132e−36
LINC00526-cg20134241	− 0.516	3.403e−29
LINC00526-cg20757519	− 0.518	2.187e−29
LINC00526-cg21311023	− 0.571	1.296e−36
LINC00526-cg26998900	− 0.502	2.023e−27
LURAP1-cg24542714	− 0.544	9.477e−33

## Discussion

Urothelial carcinoma is generally classified as non-muscle-invasive bladder cancer (NMIBC) or muscle-invasive bladder cancer (MIBC). The standard treatment for NMIBC is transurethral resection, and the universal treatment for MIBC is radical cystectomy, but a considerable number of NMIBC patients (50% to 80%) have tumour recurrence [1, 2]. Pathological staging is a key factor in current clinical decision making and prognosis of BC; nevertheless, the clinical outcomes of patients with the same stage often differ, indicating that the current staging system is not sufficient to reflect biological heterogeneity, and accurately determining the prognosis of patients is challenging. A new prognostic evaluation model based on molecular entities could guide individualized treatment and improve the therapeutic effect.

DNA methylation is an epigenetic modification that affects the interaction between DNA and regulatory factors, which, in turn, regulates gene expression [25]. Hypermethylation inhibits gene expression, while hypomethylation promotes gene expression. In addition, the DNA methylation status is faithfully inheritable through cell division but also revisable, it plays a very important role in the dynamic regulation of expression. Numerous studies based on either a genome-wide view or a gene-specific view have demonstrated that DNA methylation drives abnormal gene expression and is a crucial factor in the development and progression of tumours [26]. Therefore, the methylation profiles of methylation-driven genes in tumour patients could serve as potential biomarkers [27]. This phenomenon in BC patients is extensive, and many genes have been suggested to be factors involved in pathogenesis and are used as diagnostic and prognostic biomarkers [28, 29]. Our study provides a comprehensive view of methylation-driven genes in BC, and a prognosis model based on the methylation profile of six genes was developed and has implications for both basic research and clinical applications.

We identified a cohort of 167 methylation-driven genes in BC. The functional annotation demonstrated that these genes are widely scattered in diverse biological processes and pathways ranging from signal transduction, gene regulation, and development to metabolism and cell structure. These results demonstrate that DNA methylation is involved in the dysregulation of genes with distinct functions and suggest possible mechanisms by which DNA methylation is functionally linked to outcomes in BC patients.

Six genes (IDH2, GSTM2, LURAP1, ARHGDIB, LINC00526, and ARL14) with methylation profiles closely related to survival were selected by a LASSO Cox regression. Based on their methylation level and coefficients with survival, a prognostic model was developed. The verification of this model in the whole patient set and subsets grouped by either clinical or molecular characteristics showed that the low-risk group has a better survival status. The AUC of the ROC curve of the whole cohort based on this model was 0.698 at 3 years of OS.

For further potential application of this model in clinical work, a nomogram was generated. The nomogram integrates multiple predictors and simplifies the statistical prediction model to the probability of outcome events; thus, the survival probability of individual patients can be calculated. The predicted survival rate is close to the actual survival situation (C-index: 0.694), and the nomogram has a prediction effectiveness similar to that of the ROC curve. These results indicate the excellent predictive ability of this model in the prognosis of BC patients.

The six genes included in the model were further analysed individually. The hypomethylation of IDH2 and hypermethylation of ARL14 were associated with poor prognosis, and a high expression matched hypomethylation of ARHGDIB and ARL14 was meaningfully correlated with better prognosis. Further analysis of the methylation sites showed that the hypermethylation of 5 sites in LURAP1 and GSTM2 is associated with better prognosis, and the hypermethylation of another 9 sites in ARHGDIB, LINC00526 and ARL14 is associated with poor prognosis in BC. Additionally, the methylation levels at several methylation sites were correlated with the expression levels of the associated genes, all with negative correlations, indicating that these individual methylation sites alone contributed to expression regulation.

The methylation levels of these six genes contributed to the risk score of this model either positively or negatively. Some of this contribution could be functionally explained by previous studies, but the remainder lacks explanation, as information regarding the role of these genes in cancer is very limited.

The methylation levels of ARHGDIB, LINC00526 and ARL14 are positively related to poor survival. ARHGDIB (Rho GDP dissociation inhibitor GDI beta), which is also known as RhoGDI2, is a member of the guanine nucleotide dissociation inhibitor (GDI) family [30]. Mounting evidence suggests that the reduced expression of ARHGDIB is associated with the development of several types of cancer and that its hypermethylation contributes to its reduced expression [31]. The CpG islands of ARHGDIB were relatively hypermethylated in cases of ovarian cancer relapse after chemotherapy [32]. Huang et al. [33] demonstrated that ARHGDIB is significantly associated with OS in lung cancer patients. In BC, the reduced expression of ARHGDIB is associated with shorter disease-free survival time [34–36]. In our study, the methylation level matched gene expression analysis of ARHGDIB, and the analysis of CpG sites showed that hypomethylation in the ARHGDIB gene is associated with better survival. Our result is consistent with the results of previous studies. LINC00526 is a long intergenic non-protein-coding RNA, and one study has demonstrated that it suppresses glioma progression [37]. ARL14 (ADP Ribosylation Factor Like GTPase 14) is a protein-coding gene that participates in GTP binding and signal transduction [38]. However, information regarding the role of ARL14 in cancer is lacking.

The methylation level of IDH2, LURAP1 and GSTM2 is negatively related to poor survival. IDH2 is a protein-coding gene. The function of IDH2 in cancer has been relatively well documented. Li et al. [39, 40] found that IDH2 promotes lung cancer cell growth and serves as a novel therapeutic target in lung cancer. Mutations of IDH2 are frequently observed in acute myeloid leukaemia [41], colon cancer [42, 43], and gliomas [44], causing alterations in metabolism and DNA methylation; these mutations could represent a possible mechanism of tumorigenesis [44] and provide potential avenues for therapeutic intervention. We found that hypermethylation in IDH2 is associated with a better prognosis in BC patients. In our study, the relationship among GSTM2, LURAP1 and prognosis showed similar characteristics to IDH2. Hypermethylation at 3 sites in GSTM2 and 2 sites in LURAP1 is correlated with a better prognosis. GSTM2 is a subtype of glutathione S-transferase (GSTs) that performs functions such as eliminating free radicals and is involved in cell protection and the regulation of cell growth. Consistent with our findings, Kresovich et al. [45] found that a high methylation level in the GSTM2 promoter could be involved in ER/PR-negative breast cancer progression. Ashour et al. [46] proved that the epigenetic silencing of GSTM2 is a common phenomenon in prostate cancer that could be used as a molecular marker for diagnosis.

To the best of our knowledge, these six genes have not been previously studied as a prognostic model in BC patients. Further verification of this model in other types of clinical specimen, such as urine sediment cells and circulating tumour cells from BC patients, could provide more information regarding its potential clinical application. For urologists, accurate prognostic assessments are critical for selecting the optimal treatment. Our nomogram is a predictive model that combines gene information and clinical factors to provide a prognostic indication for clinicians.

However, this study also has certain limitations. First, this is a retrospective study, and the application of this model requires further verification by increasing the sample size and performing prospective studies. Second, the treatments that the patients have received are highly heterogeneous and incomplete, thus we could not include this information in our analysis. Improving these aspects for future in-depth studies could further increase the persuasiveness of these results.

In summary, we screened methylation-driven genes in BC, and a six-gene model was constructed based on methylation profiles. This model was validated in groups with different disease characteristics and could be expected to serve as a predictive tool for clinical outcomes and guide personalized anticancer treatment. In addition, we analysed the relationships between individual CpG islands associated with these six genes and survival, which may provide important bioinformatic clues for mechanistic research related to the development and progression of BC.

## Conclusion

Based on public data from the TCGA database, we used MethylMix in R and a LASSO Cox analysis to screen methylation-driven genes associated with prognosis in BC patients. A prediction model based on methylation of six genes (IDH2, GSTM2, LURAP1, ARHGDIB, LINC00526, ARL14) was constructed. The verification in different subgroups demonstrated the validity and consistency of the model. A nomogram was further constructed to predict the likelihood of OS. The ROC curve, nomogram calibration plots and comprehensive survival analysis of each gene revealed that this model is an effective predictive model that can be used as a prognostic marker in BC patients. These results indicate that methylation detection may be an important means to help establish a new evaluation system for prognosis and act as a therapeutic target for antitumour drug development.

## Supplementary information

**Additional file 1: Table S1.** Expression level of 167 driven genes.

**Additional file 2: Table S2.** Methylation level of 167 driven genes.

**Additional file 3: Table S3.** Metascape functional analysis results.

**Additional file 4: Table S4.** Clinical signatures, mRNA types and mutational signatures of all patients.

**Additional file 5: Figure S1.** Kaplan–Meier curves based on mRNA types and mutational signatures.

**Additional file 6: Figure S2.** Nomogram of six genes.

**Additional file 7: Figure S3.** Correlation between methylated sites and gene expression.

## Data Availability

All data generated or analysed in this study are included in this published article.

## Abbreviations

LASSO: least absolute shrinkage and selection operator
BC: bladder cancer
GO: gene ontology
KEGG: Kyoto Encyclopedia of Genes and Genomes
TCGA: The Cancer Genome Atlas
OS: overall survival
BP: biological process
MF: molecular function
C-index: concordance index
NMIBC: non-muscle-invasive bladder cancer
MIBC: muscle-invasive bladder cancer
CI: confidence interval
HR: hazard ratio
