# Supplementary figures and images for: A six-gene prognostic model predicts overall survival in bladder cancer patients
Source: Cancer Cell Int. 2019 Sep 5;19:229. doi: 10.1186/s12935-019-0950-7 (PMC6729005; doi:10.1186/s12935-019-0950-7)

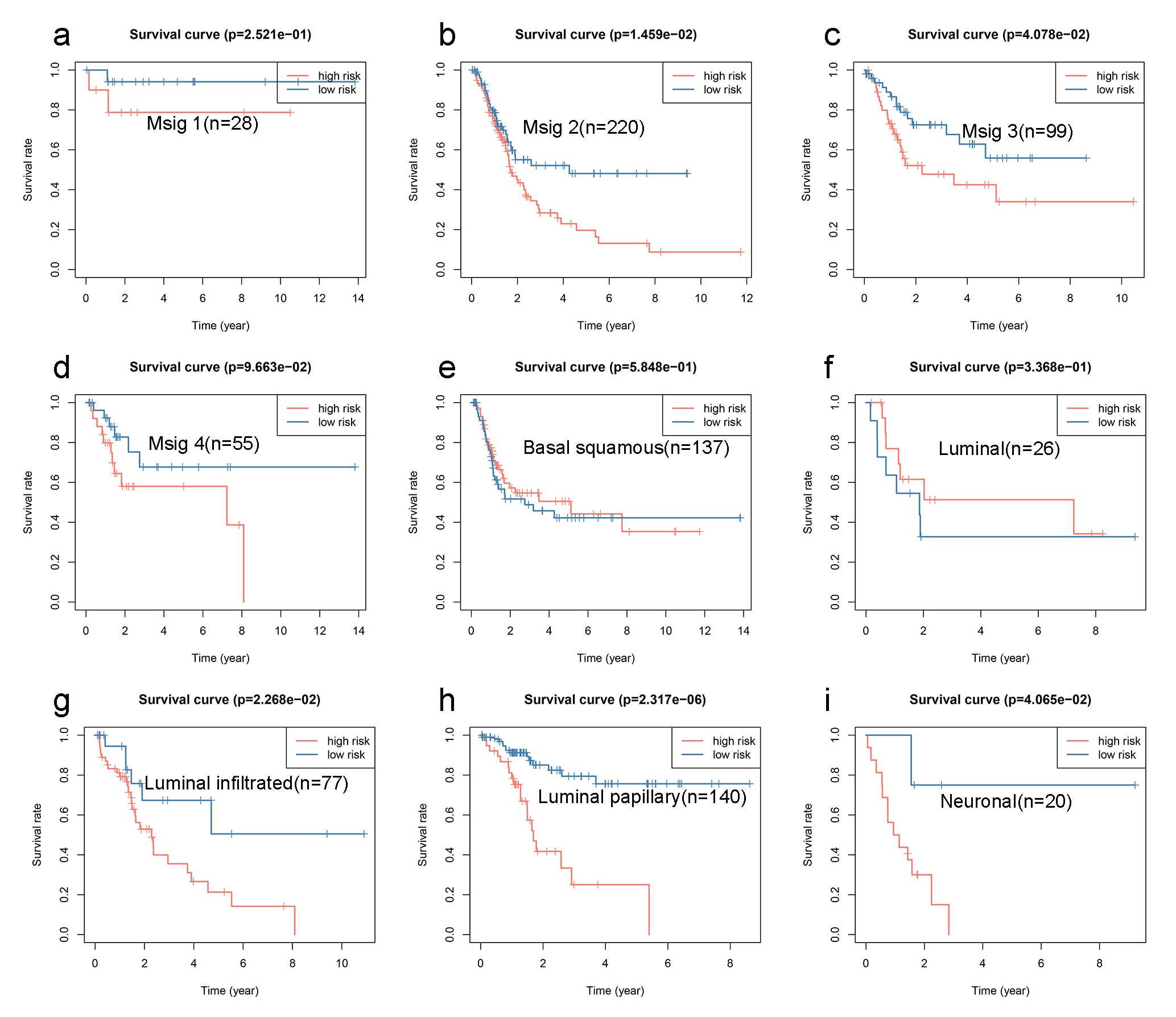

Supplement: Supplementary file 5 — Additional file 5: Figure S1. Kaplan–Meier curves based on mRNA types and mutational signatures. [file 12935_2019_950_MOESM5_ESM.tif]

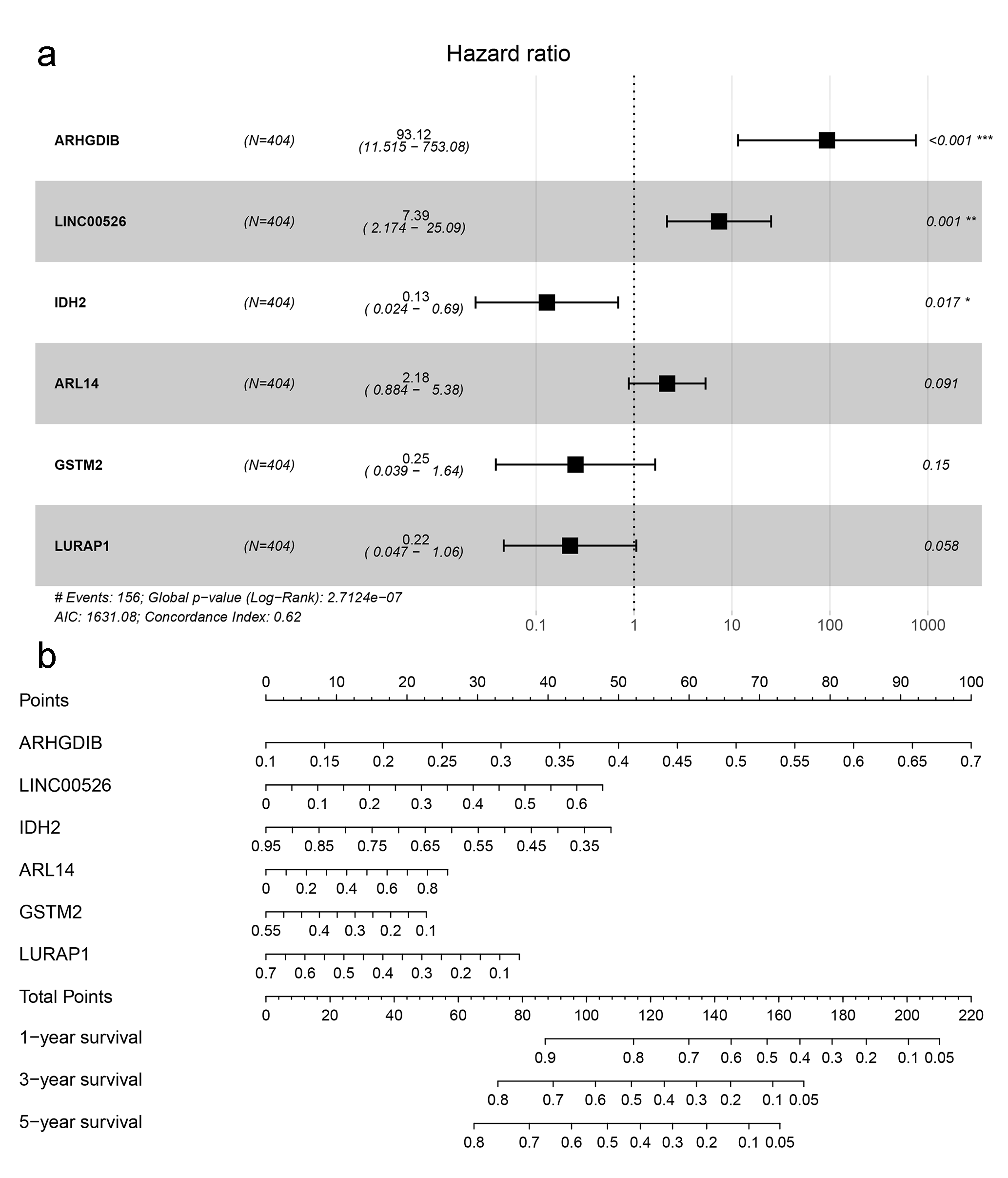

Supplement: Supplementary file 6 — Additional file 6: Figure S2. Nomogram of six genes. [file 12935_2019_950_MOESM6_ESM.tif]

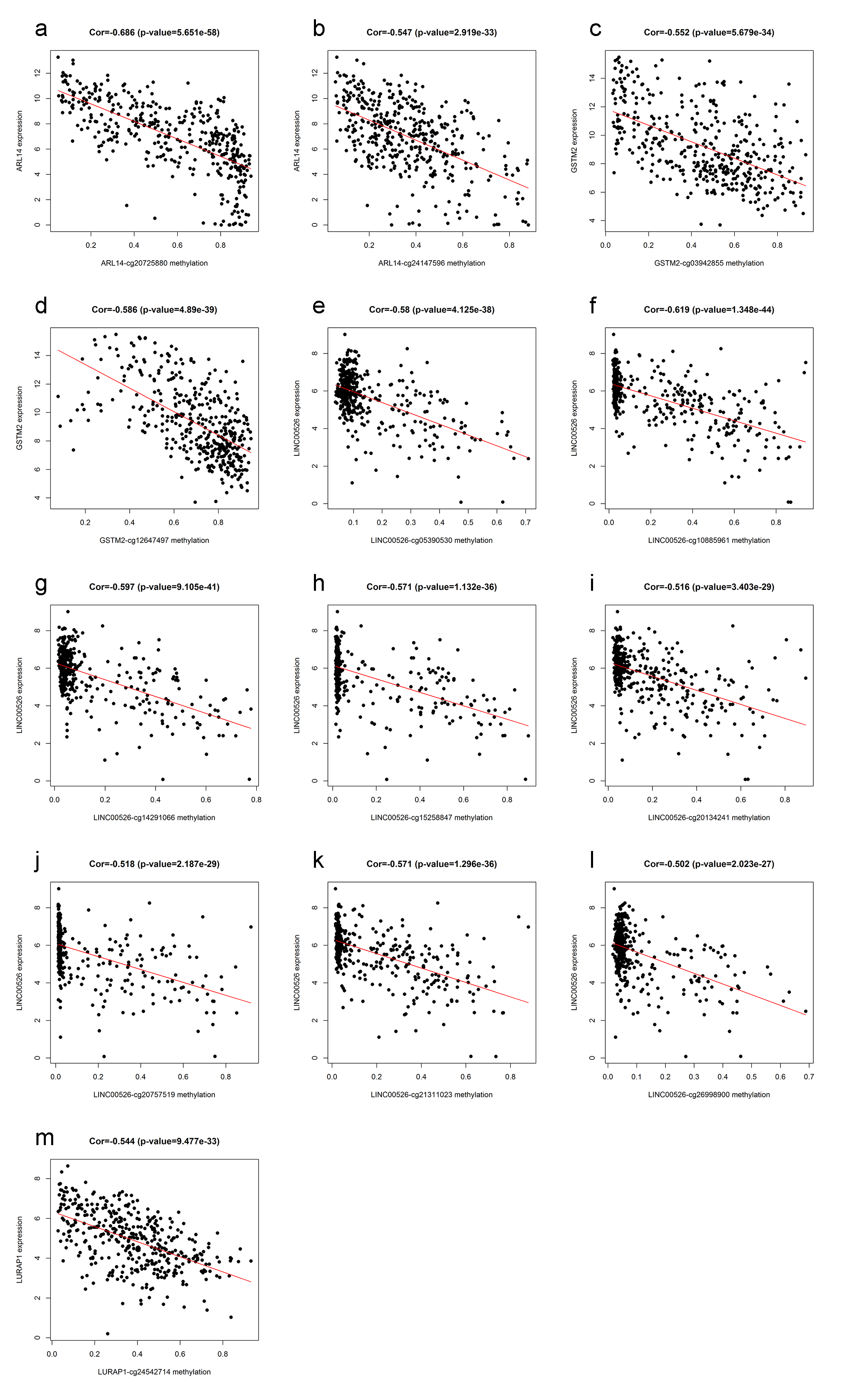

Supplement: Supplementary file 7 — Additional file 7: Figure S3. Correlation between methylated sites and gene expression. [file 12935_2019_950_MOESM7_ESM.tif]
